# Consensus Paper: Neuroimmune Mechanisms of Cerebellar Ataxias

**DOI:** 10.1007/s12311-015-0664-x

**Published:** 2015-03-31

**Authors:** Hiroshi Mitoma, Keya Adhikari, Daniel Aeschlimann, Partha Chattopadhyay, Marios Hadjivassiliou, Christiane S. Hampe, Jérôme Honnorat, Bastien Joubert, Shinji Kakei, Jongho Lee, Mario Manto, Akiko Matsunaga, Hidehiro Mizusawa, Kazunori Nanri, Priya Shanmugarajah, Makoto Yoneda, Nobuhiro Yuki

**Affiliations:** 1 Department of Medical Education, Tokyo Medical University, Tokyo, Japan; 2 Department of Haematology, Nil Ratan Sircar Medical College, 138 A J C Bose Road, Kolkata, 700014 West Bengal India; 3 Matrix Biology &Tissue Repair Research Unit, School of Dentistry, College of Biomedical and Life Sciences, Cardiff University, Cardiff, Wales UK; 4 Department of General Medicine, College of Medicine & Sagore Dutta Hospital, 578 B T Road, Kamarhati-Kolkata, 700056 West Bengal India; 5 Academic Department of Neurosciences, Royal Hallamshire Hospital, Sheffield, UK; 6 School of Medicine, University of Washington, 850 Republication, Seattle, WA 98109 USA; 7 University Lyon 1, University Lyon, Rue Guillaume Paradin, 69372 Lyon Cedex 08, France; 8 INSERM, UMR-S1028, CNRS, UMR-5292, Neuro-Oncology and Neuro-Inflammation Team, 7, Lyon Neuroscience Research Center, Rue Guillaume Paradin, 69372 Lyon Cedex 08, France; 9 National Reference Centre for Paraneoplastic Neurological Diseases, Hospices Civils de Lyon, Hôpital Neurologique, 69677 Bron, France; 10 Hospices Civils de Lyon, Neuro-oncology, Hôpital Neurologique, 69677 Bron, France; 11 Tokyo Metropolitan Institute of Medical Science, Tokyo, Japan; 12 Unité d’Etude du Mouvement, FNRS, Neurologie ULB-Erasme, 808 Route de Lennik, 1070 Brussels, Belgium; 13 Department of Neurology, University of Fukui Hospital, Fukui, Japan; 14 National Center of Neurology and Psychiatry, Tokyo, Japan; 15 Department of Neurology, Tokyo Medical University Hachioji Medical Center, Tokyo, Japan; 16 Academic Department of Neurosciences, Royal Hallamshire Hospital, Sheffield, UK; 17 Faculty of Nursing and Social Welfare Sciences, Fukui Prefectural University, Fukui, Japan; 18 Departments of Medicine and Physiology, Yong Loo Lin School of Medicine, National University of Singapore, Singapore, Singapore

**Keywords:** Cerebellar ataxias, Anti-GAD antibodies, Hashimoto’s encephalopathy, Primary autoimmune cerebellar ataxia, Gluten ataxia, Systemic lupus erythematosus, Miller Fisher syndrome, Paraneoplastic cerebellar degeneration

## Abstract

In the last few years, a lot of publications suggested that disabling cerebellar ataxias may develop through immune-mediated mechanisms. In this consensus paper, we discuss the clinical features of the main described immune-mediated cerebellar ataxias and address their presumed pathogenesis. Immune-mediated cerebellar ataxias include cerebellar ataxia associated with anti-GAD antibodies, the cerebellar type of Hashimoto’s encephalopathy, primary autoimmune cerebellar ataxia, gluten ataxia, Miller Fisher syndrome, ataxia associated with systemic lupus erythematosus, and paraneoplastic cerebellar degeneration. Humoral mechanisms, cell-mediated immunity, inflammation, and vascular injuries contribute to the cerebellar deficits in immune-mediated cerebellar ataxias.

## Introduction (H. Mitoma)

### Historical Scope

More and more evidence suggest that cerebellar ataxias (CAs) in some patients develop through immune-mediated mechanisms. The involvement of immune-mediated pathomechanisms in the etiology of CAs was first proposed in the context of multiple sclerosis (MS). In a well-cited lecture delivered in 1868 [1], J.M. Charcot described the relapsing and remitting clinical course and the pathology of demyelination. The same lecture also described the presence of cerebellar symptoms, such as intention tremor, scanning speech and nystagmus, in addition to those of optic neuritis and paralysis. These cerebellar symptoms, which were subsequently termed the Charcot’s triad, were considered to provide evidence for lesions in supraspinal structures. Although CAs rarely occur in isolation during the first attack (the reported incidence of CAs based on symptoms at presentation was about 15 %), they are common in established MS [2]. Based on the results of experimental allergic encephalomyelitis, the following cell-mediated mechanisms responsible for myelin damage have been proposed [2]: (1) Lymphocytes activated by antigens or those that present antigens, enter the blood brain barrier, where they can recruit other immune cells, and (2) lymphocyte-induced myelin damage is mediated through the actions of certain substances, e.g., tumor necrosis factor in plaques.

In MS, immune-mediated demyelination can occur in any area of the central nervous system (CNS) white matter and, consequently, in addition to ataxia there is a wide spectrum of neurological symptoms. However, in the last years, many authors described patients with the cerebellum as the sole target of autoimmunity, such as paraneoplastic cerebellar degeneration.

The first cases of paraneoplastic cerebellar degeneration associated with an ovary carcinoma were reported in 1919 by Brouwer [3]. This was followed by the identification of other cases associated with different kind of cancers such as breast or lung carcinoma and the description of specific autoantibodies (anti-Yo) in patients with CAs and gynecological cancer [4]. Since then, various autoantibodies have been identified depending on neoplasma or associated extracerebellar symptoms [5]. Furthermore, seronegative types of paraneoplastic cerebellar degenerations have also been reported [5]. The association of cerebellar autoantibodies with CAs suggests that autoimmunity triggered by the neoplasm results in the development of CA-related symptoms.

In contrast to the well-established concept of paraneoplastic cerebellar degeneration, the clinical entity of nonparaneoplastic immune-mediated CAs was only established recently [6–8]. In the 1980s, these cases were reported in association with autoantibodies, and three clinical entities have been established so far, based on the kind of the associated antibodies (Abs): CA with Abs to glutamic acid decarboxylase (GAD), CA with Abs to thyroid tissue (cerebellar type of Hashimoto’s encephalopathy), and CA with Abs to gliadin (gluten ataxia). The features characterizing these entities are as follows: (1) positivity for antibodies in the serum or cerebrospinal fluid (CSF); (2) mild or no atrophy of the cerebellum on magnetic resonance imaging (MRI) at the early stage; and, (3) improvement of CAs, at least in part, by immunotherapy or in the case of gluten ataxia strict adherence to a gluten-free diet. In addition to these three subtypes, the concept of primary autoimmune cerebellar ataxia (PACA) was recently proposed as an additional immune-mediated CA [9]. In PACA, the cerebellum is a primary target of autoimmunity and is sometimes associated with cerebellar autoantibodies. Recently, the association with Homer 3, ARHGAP26, and ITPR1 antibodies was also reported [10–12].

### The Concept of Brain Autoimmune Diseases

In the last 10 years, different authors have identified that, in some patients with encephalitis, the disorders are immune mediated. These patients may present complex neuropsychiatric symptoms such as deficits of memory, cognition, psychosis, seizures, abnormal movements, or coma. These disorders affect mainly young women, but also men and children, and are potentially lethal but curable if promptly recognized and treated by immunomodulators. The most important discovery is that these disorders occur in association with autoantibodies to extracellular epitopes of receptors or proteins involved in synaptic transmission and plasticity, such as voltage-gated potassium channel complex (LGI1, CASPR2) [13, 14], AMPA [15], NMDA [16] and GABA_B_ [17] receptors. Some of these patients develop a tumor and the syndrome can be qualified as paraneoplastic [18], but most of the cases are idiopathic. Interestingly, many arguments suggest that associated autoantibodies may play a direct role in the pathomechanisms of the encephalitis. For example, autoantibodies directed to AMPA and NMDA receptors decrease the numbers of these cell-surface receptors (internalization), which could lead to behavioral deficits [19–21]. Furthermore, autoantibodies to AMPA receptor also act as agonists and increase cell excitability [22]. The above findings suggest that autoimmune mechanisms play a role in the aetiopathology of such encephalitis.

Interestingly, the cerebellum and the hippocampus are two brain regions preferentially targeted by autoimmunity. However, at this stage of our knowledge, immune-mediated CAs appear to have more heterogeneous or diverse features with respect to autoimmune pathogenesis, compared with autoimmune limbic encephalitis, which shows somewhat uniform features with regard to the nature of antigens (localization and role in cell physiology), induction of autoimmunity, and perhaps, significance of autoantibodies.

### Significance and Classification

Although immune-mediated CAs can be characterized as *treatable ataxias*, immunotherapy seems to be effective only at the early stages of the disease. On the other hand, accumulating evidence suggests a higher than expected incidence of immune-mediated CAs among sporadic CAs. Prospective studies by Hadjivassiliou et al. [9] in the UK showed that the prevalence of immune-mediated CAs was 32 % in a group of 320 patients with sporadic ataxia (gluten ataxia 27 %, paraneoplastic cerebellar degeneration 3 %, and anti-GAD-Abs associated CA 3 %). Taken together, clinicians are now required to establish the diagnosis of immune-mediated CAs and to initiate immunotherapy at an early stage. For selection of adequate therapeutic strategies, a good understanding of the pathomechanisms of CAs is necessary.

When autoimmunity targets the cerebellum or its related structures, immune-mediated CAs are classified according to the trigger of autoimmunity [9] (Table 1). Immune-mediated CAs in which autoimmunity is not clearly triggered by another disease include anti-GAD-Abs associated CA, cerebellar type of Hashimoto’s encephalopathy, and PACA. On the other hand, immune-mediated CAs in which autoimmunity is triggered by another disease include gluten ataxia, Miller Fisher syndrome, and paraneoplastic cerebellar degeneration. These diseases are triggered by gluten sensitivity, infection, or neoplasm, respectively.

**Table 1:** The classification of immune-mediated cerebellar ataxias

1. Autoimmunity that mainly targets the cerebellum^a^ or its related structures^b^:	
Cerebellar autoimmunity not triggered by another disease:	
Anti-GAD Abs associated cerebellar ataxia	
Cerebellar type of Hashimoto’s encephalopathy	
Primary autoimmune cerebellar ataxia	
Others	
Cerebellar autoimmunity triggered by another disease or condition:	
Gluten ataxia	(gluten sensitivity)
Acute cerebellitis	(infection)
Miller Fisher syndrome	(infection)
Paraneoplastic cerebellar degenerations	(neoplasm)
2. Autoimmunity that simultaneously targets various parts of the CNS:	
Multiple sclerosis	
Ataxia in the context of connective tissue diseases such as SLE	

^a^When cerebellar ataxias are sole or main symptoms, the cerebellum is presumed to be the main target of autoimmunity

^b^For example, involvement of the proprioceptive spinocerebellar pathway is assumed in Miller Fisher syndrome

^c^Paraneoplastic patients are exceptional

### Aim of this Consensus Paper

The objective of this consensus paper is to summarize the clinical features of each immune-mediated CA entity in which the target of autoimmunity is the cerebellum or its related structures, and address their immune-mediated pathomechanisms. To this aim, we prepared contributions from international experts involved in research on various aspects of immune-mediated CAs, providing key concept in each subtopic. For comparative understanding of key concepts of clinical features and pathomechanisms of each subtype of immune-mediated CA, the experts have provided summaries on the current thinking about immune-mediated CAs. Some newly emerging entities such as Hashimoto’s encephalopathy and PACA have been focuses of discussions and do not have widely accepted diagnostic criteria. In addition, diverse immune-mediated pathomechansms have not been clarified yet. In this sense, further research should be performed. However, we believe that the present consensus will be valuable to clarify the topic and trigger novel research.

## Cerebellar Ataxia with Glutamic Acid Decarboxylase Autoantibodies (B. Joubert and J. Honnorat)

Glutamic acid decarboxylase (GAD) is the rate-limiting enzyme for the production of GABA (γ-aminobutyric acid), the main inhibitory neurotransmitter in the CNS. GAD is expressed in CNS GABAergic neurons and in the pancreatic islet β-cells and exists as two isoforms, GAD65 and GAD67 [23]. The soluble isoform GAD67, mainly expressed in the cytoplasm of neurons, is suggested to regulate the basal levels of GABA [24], while the shorter form GAD65 is associated with the cell membrane at nerve terminals and is involved both in the synthesis of GABA and its exocytosis at inhibitory synapses [25].

Anti-GAD65 antibodies (GAD-Ab) were first described in type 1 diabetes mellitus (T1DM) patients, and are considered as a biological marker of this disease. GAD-Ab have also been reported in some patients with neurological diseases, such as stiff person syndrome (SPS), cerebellar ataxia, limbic encephalitis, abnormal eye movements, progressive encephalomyelitis with rigidity and myoclonus and refractory epilepsy [26].

Cerebellar ataxia with GAD-Ab was initially described in few case reports [27, 28] and in a series of 14 patients [6]. Although rare, this syndrome is now well established [26]. This disease affects mostly women in their sixth decade. Cerebellar ataxia installs either insidiously or subacutely [6, 29] and tends to progress continuously over time. Symptoms include mainly static ataxia, dysarthria, and nystagmus. Furthermore, cerebellar ataxia may coexist with SPS [29, 30], peripheral neuropathy, limb stiffness [6], and myasthenia gravis [28]. A personal or familial history of other autoimmune diseases, such as T1DM, hemolytic anemia or thyroiditis is frequent [6], while a paraneoplastic origin is exceptional [31]. Brain MRI generally shows cerebellar atrophy, exclusively in patients with cerebellar ataxia without SPS [6, 30]. CSF analysis usually displays oligoclonal bands and intrathecal synthesis of GAD-Ab with normal cellularity and protein rate [6, 26]. Disease severity, phenotype, and response to treatment are not correlated with serum or CSF GAD-Ab titers.

While SPS with GAD-Ab tends to respond favorably to immunotherapy, cerebellar ataxia with GAD-Ab is usually associated with a poor prognosis, most patients remaining significantly disabled. Large-scale randomized studies are lacking to determine the optimal therapeutic strategies in GAD-Ab cerebellar ataxias. If corticosteroids are often disappointing, intravenous immunoglobulins (IvIg) may have a beneficial effect in some patients [29, 32].

Pathophysiology and mechanisms of neuronal dysfunctions observed in patients with GAD-Ab are unknown. Some studies suggest that, in contrast to GAD-Ab associated with T1DM without neurological sign, GAD-Ab from patients with neurological symptoms could be more than merely a marker of autoimmunity and play a direct role in the development of the disease [33, 34]. Indeed, intra-cerebellar or intraventricular administration of IgGs from patients with GAD-Ab and SPS or cerebellar ataxia blocks neuronal functions and impairs synaptic regulation [33, 35]. Furthermore, mice immunization against GAD65 leads development of GAD-Ab and GABAergic neuronal loss [36]. Interestingly, the effect of GAD-Ab on neuronal functions seems to differ accordingly to the neurological symptoms observed in patients. Indeed, GAD-Ab from patients with cerebellar ataxia, contrarily to GAD-Ab from SPS patients, do not inhibit GAD enzymatic activity in vitro [24, 34]. Moreover, electrophysiological and neurotransmitter levels alterations observed in rats treated with intra-cerebellar and para-spinal infusions with IgGs from GAD-Ab positive SPS are not observed with IgGs from patients with GAD-Ab positive cerebellar ataxia [34, 37]. On the opposite, IgGs from patients with GAD-Ab positive cerebellar ataxia are able to impair membrane turnover in GABAergic neurons, suggesting an interference with GABA-containing vesicles exocytosis [34]. These differences could be explained by distinct epitope specificities recognized by GAD-Ab depending on the patients. Even if GAD-Ab production is polyclonal in all patients, GAD-Ab recognize different epitopes according to whether the associated syndrome is T1DM, SPS or cerebellar ataxia [34]. The importance of such GAD-Ab epitope specificities in the disruption of neuronal functions is discussed in the next section. However, at this time, even if many arguments suggest a pathophysiological role of GAD-Ab, there is no clear demonstration that GAD-Ab alone are able to provoke SPS or cerebellar ataxia. Some authors suggested that other associated uncharacterized autoantibodies might also play a role [36]. Cellular immunity could also play a major role in patients with cerebellar ataxia [36]. Indeed, peripheral T cells from patients with GAD-Ab and cerebellar ataxia, but not SPS patients, increase their production of INF-γ following exposition to GAD, suggesting a Th1 orientation of those cells, a profile known to favor cytotoxicity [38].

To conclude, GAD65 is a particularly antigenic protein, and GAD-Ab can be observed in different kinds of autoimmune disease or neurological syndromes where they probably do not have the same clinical significance. GAD-Ab positivity must not be interpreted alone, but in consideration with all other potentially associated autoantibodies [29]. In the case of neurological syndrome, intrathecal synthesis of GAD-Ab is a strong argument for a potential role of these autoantibodies, especially if the titer is low. Further works will be necessary to understand the exact mechanisms of cerebellar ataxia in patients with GAD-Ab.

## Pathogenic Roles of GAD65 Antibodies (M. Manto and CS. Hampe)

Unlike the isoform, GAD67, which is located in the cytoplasm, is constantly active, and produces a steady basal level of GABA, the isoform GAD65 is mainly found associated with synaptic vesicles, undergoes autoinactivation with at least 50 % of cellular GAD65 in the inactive apoenzyme form, and generates pulses in GABA production when circumstances require a rapid synthesis and release [39, 40]. The enzyme is necessary for the fine tuning of GABA-concentrations in the vesicles and is also located on the cytosolic face of GABAergic vesicles in association with vesicular GABA transporter (VGAT) [41] and other anchoring proteins, such as NAP-22 [42]. The enzyme is involved both in GABA transport into synaptic vesicles and in controlling synaptic release of the neurotransmitter [35]. In GAD65 knockout mice, the quantal size and frequency of GABA-mediated miniature inhibitory postsynaptic currents are normal, but evoked GABAergic inhibitory post synaptic currents are reduced [43] possibly contributing to the increased anxiety and propensity for epilepsy observed in the animals [44]. It is currently presumed that the effects of GAD-Ab on GABA levels are mediated by a direct inhibition of the enzymatic activity of GAD65 and an impairment of exocytosis of GABAergic vesicles.

The spectrum of autoimmune neurological deficits associated with GAD-Ab includes not only SPS, but also rare cases of limbic encephalitis, epilepsy, and progressive cerebellar ataxia [26, 45, 46]. Common comorbidities are T1DM and autoimmune polyendocrine syndrome. Intrathecal production of GAD-Ab has been reported in patients with stiff person syndrome (SPS) [47], and GAD-Ab are typically found in peripheral blood with lower concentrations in the CSF [48]. GAD-Ab titers do not necessarily correlate with disease severity and cases with lower GAD-Ab levels have been reported recently in ataxic patients [49]. Recent studies suggest that the epitope specificity of GAD-Ab determine the pathogenicity of the autoantibody (discussed in detail below). The pathophysiology of SPS includes decreased concentrations of GABA in the brain and CSF [50] and inflammation in the CNS [45]. Cell loss has been demonstrated in ataxic patients, with depletion of Purkinje neurons, Bergmann gliosis, and a relative sparing of basket cells [51].

The direct pathogenic effects of GAD-Ab are still a matter of debate [52]. Among the arguments against a direct pathogenic role are: (1) the intracellular location of GAD65 making it unlikely to illicit an antibody response, (2) the inaccessibility of intraneuronal GAD65 to its antibodies since neurons have often been considered as “impermeable” to immunoglobulins, (3) the possibility that other Ab—or other undetected substances—present concomitantly in the serum might be pathogenic. For the first issue, it can be argued that GAD65 could be exposed during exocytosis and thus provide a direct opportunity for an interaction between Ab and antigen [53, 54]. For the second issue, it has been demonstrated that Purkinje neurons (one key-target population of GAD-Ab in the cerebellum) in rat organotypic cultures incorporate and clear both host and non host immunoglobulin, even if the antibodies do not recognize Purkinje cell antigens [55]. IgG within Purkinje cells accumulates in cell nuclei and the cytoplasm. Moreover, kappa and lambda light chains in human Purkinje neurons have been detected in a patient with multiple myeloma [56]. The Purkinje neurons might thus be an exception to the rule of neuronal exclusion of antibodies. However, the demonstration of uptake of a different SPS-associated autoantibody—directed to amphiphysin—by hippocampal neurons [57] may indicate that neuronal uptake of immunoglobulin is more frequent than previously recognized. The third issue seems rather unlikely to occur with the use of monoclonal GAD-Ab. Still, SPS is an example of a disorder where autoantibodies other than GAD-Ab including anti-amphiphysin, anti-GABA receptor-associated protein (GABARAP) and anti-gephyrin Ab, can cause a similar phenotype, with each of these autoantigens being associated with GABA-mediated neurotransmission [39].

Recent studies underline the importance of epitope specificity of GAD-Ab in the pathophysiology of GAD65-related neurological disorders. As discussed in the previous section of this publication, GAD-Ab are heterogeneous and target distinct epitopes of the enzyme. GAD-Ab in neurological disorders recognize epitopes that differ significantly from those found in patients with T1DM [58]. Importantly, GAD-Ab present in SPS patients typically inhibit GAD65 enzyme activity, a characteristic not observed for GAD-Ab found in patients with T1DM [48]. Intrathecal passive transfer of monoclonal GAD-Ab representing an epitope specificity characteristic for SPS or purified immunoglobulin from sera from GAD-Ab-positive SPS patients impair spinal cord activity, induce a SPS-like syndrome with motor hyperexcitability, change behavior, and cognitive operations [34, 35, 37, 59, 60]. GAD-Ab from ataxic patients suppress the release of GABA and depress the inhibitory transmission long-lasting time course [61]. In contrast, monoclonal GAD-Ab representing an epitope specificity characteristic for T1DM do not affect the activity of the enzyme [48] and do not interfere with exocytosis [59]. This epitope-dependent effect of neurological deficits induced by in vivo administration of monoclonal GAD-Ab may explain why patients with T1DM do not show neurological signs [33, 34, 37, 62].

In summary, despite accumulating in vitro and in vivo findings arguing in favor of a direct pathogenic effect of GAD-Ab, the debate is still alive. The unequivocal pathophysiological demonstration is missing, at a moment where the critical roles of antibodies for the diagnosis of GAD-Ab-related disorders have been clearly accepted by the scientific community [63].

## Cerebellar Type of Hashimoto’s Encephalopathy-Diversity of Clinical Profiles (A. Matsunaga and M. Yoneda)

### Hashimoto’s Encephalopathy

Hashimoto’s encephalopathy (HE) is an autoimmune treatable encephalopathy associated with chronic thyroiditis, and is distinct from myxedema-encephalopathy associated with hypothyroidism [7, 64]. The clinical entity and nosology of HE have been debated and the reality of the illness questioned because of no evidence establishing a direct relationship between thyroid antibodies and encephalopathy and the high prevalence of anti-thyroid antibodies in the normal population. However, accumulated case reports support the proposal of this autoimmune treatable disease, HE, based on response to immunotherapy, pathologic findings of brain tissue and presence of some autoantibodies [65].

HE patients present with a variety of neuropsychiatric symptoms such as consciousness disturbance, psychosis, seizures, cognitive impairment, involuntary movements, and cerebellar ataxia [7, 66]. Some diagnostic criteria of HE have been proposed as a combination of neurological and/or psychiatric symptoms, elevated serum anti-thyroid antibodies [Abs] (anti-thyroglobulin [TG] Abs and/or anti-thyroid peroxidase [TPO] Abs) and responsiveness to steroid, associated in part with abnormal EEG, normal brain imaging and elevated proteins in CSF [7, 66]. Several autoantibodies relevant to HE have recently been identified [65, 67, 68]. We discovered serum autoantibodies against the NH_2_-terminal of α-enolase (anti-NAE Abs) in patients with HE using a proteomics method [65, 67]. The anti-NAE Abs demonstrates high specificity for HE, and can be a useful serological diagnostic marker for HE [65, 67]. Although the pathogenesis of HE remains unclear, cerebral vasculitis was evident in the biopsied or postmortem brain tissue from HE patients [69, 70].

### Cerebellar Type of Hashimoto’s Encephalopathy

Cerebellar ataxia is not a rare presentation in HE [71] (28 % of patients observed by Matsunaga et al., unpublished data), and occasionally appears as an isolated or predominant symptom of HE (6 % of cases, [72]). We previously reported a patient with HE developing cerebellar ataxia [73]. To clarify the characteristics of cerebellar type of HE, we further investigated 13 patients, all of whom showed progressive cerebellar ataxia as a predominant symptom, mimicking degenerative CAs, and fulfilled the diagnostic criteria for HE mentioned above [72]. In this investigation, all patients presented with truncal ataxia as a main symptom (100 %), but nystagmus seldom appeared (17 %) [72]. Those patients who presented with anti-NAE Abs tend to respond better to immune therapy than anti-NAE Abs-negative patients [72].

Table 2 summarizes the reported cases showing cerebellar ataxia as a predominant or isolated symptom in HE, including our 13 cases reported previously [71–77]. The age of onset ranged from 38 to 84 years old, and the male/female ratio was 8 to 10. Truncal ataxia is predominant in cerebellar symptoms. The onset of cerebellar type of HE patients varied from acute to chronic. The majority of the patients had an insidious onset mimicking degenerative CAs [72, 73, 75, 77], whereas a few patients presented with acute onset mimicking strokes [74, 76]. Serum anti-TG and/or anti-TPO Abs were detected in all patients, and serum anti-NAE Abs was also detected in some Japanese patients [72, 73, 76]. Most cases described in the literatures showed elevated protein in CSF, while only one of our 13 patients demonstrated elevated protein in CSF [72]. Although these cases presented with severe cerebellar ataxia, the brain imaging showed little or no cerebellar atrophy except for one case reported by Selim and Drachman [75]. Some patients revealed diffuse slow waves on EEG as common presentation in HE [7, 65, 66]. All patients responded to immunotherapies except for one case reported by Yamamoto et al. with spontaneous remission [76]. Furthermore, most cases showed complete remission by administration of corticosteroids, few cases required immunoglobulin and/or immune-suppressants. Recurrence of ataxia usually was apparent when corticosteroids were tapered.

**Table 2:** Comparison of clinical features of nonparaneoplastic immune-mediated cerebellar ataxia

	GAD^a^, *n* (%)	HE^b^, *n* (%)	Gluten^c^, *n* (%)
Total number	58	18	Epidemiology and symptoms; 68, Therapy; 26
Number of women (%)	50/58 (86)	10/18 (56)	33/68 (49)
Mean age (years)	58 years [26–79]	53 years	48 years [14–80]
Insidious onset	38/58 (66)	10/18 (56)	Unknown
Cerebellar signs			
Nystagmus	37/58 (64)	2/18 (11)	57/68 (84)
Dysarthria	38/58 (66)	11/18 (61)	45/68 (66)
Gait ataxia	55/58 (95)	17/18 (94)	68/68 (100)
Limb ataxia	41/58 (71)	13/18 (72)	Upper 51/68 (75), Lower 61/68 (90)
Other symptoms	SPS, limb stiffness, MG: 14/58 (24)	Neuropsychiatric symptoms: 11/18 (61)	Sensorimotor axonal neuropathy: 31/68 (45), gluten-sensitive enteropathy: 16/68 (24), gastrointestinal symptoms: 9/68 (13), focal myoclonus, palatal tremor, and opsoconus^a^
Associated autoimmune diseases	45/58 (76), TDM1, thyroiditis, hemolytic anemia	Common, Thyroiditis	Common, thyroiditis, TDM1, pernicious anemia
Autoantibodies	Anti-GAD-Ab TPO, TG, ANA: 30/41 (73)	TPO, TG NAE: 9/18 (50)	Anti-gliadin Ab, TG2 Ab, TG6 Ab^b^
High proteins in CSF	Unknown	6/18 (33)	Unknown
Cerebellar atrophy on MRI	3/7 (43)	7/18 (39)	54/68 (79)
EEG	Unknown	Slow wave, 5/14 (36)	Unknown
Immunotherapy	Steroids, IVIg, oral immunosuppressants	Steroids, IVIg, oral immunosuppressants	Gluten-free diet (or IVIg for cases with gluten-free diet resistance ^c^.), mycophenolate for cases with myoclonic ataxia
Outcome			
Complete remission	0/20 (0)	7/18 (39)	0/26 (0)
Partial remission	7/20 (35)	11/18 (61)	26/26 (100)

*GAD* anti-GAD-Abs-associated CA, *HE* cerebellar type of Hashimoto’s encephalopathy, *gluten* gluten ataxia, *n* number of patients

^a^Patients with GAD antibodies and cerebellar ataxia were evaluated using the series published by Honnorat et al. [6]; Saiz et al. [26], and Ariño et al. [29]. One patient, reported in 2001, was excluded since 10 years after the publication she was found to have autosomique recessive ataxia [158]

^b^Patients with cerebellar type of HE were evaluated based on the report by Manto et al. [74, 145]; Selim et al. ([75], reported as Patient 5); Passarella et al. [71]; Yamamoto et al. [76]; Tang et al. [77], and Matsunaga et al. [72]. *TPO* anti-thyroid peroxidase Ab, *TG* anti-thyroglobulin Ab, *NAE* anti-NH_2_-terminal of α-enolase Ab

^c^Epidemiological and neurological symptoms were cited form the report of Hadjivassiliou et al. [79], and results of gluten-free diet were evaluated using the report of Hadjivassiliou et al. [85]. (a) Sarrigiannis et al. [88], Kheder et al. [89], Deconinck et al. [90]. (b) Hadjivassiliou et al. ([84, 87] and [104]), (c) Burk et al. [94]. *TG2 Ab and TG6 Ab* anti-transglutaminase 2 and 6 Abs

The pathological mechanisms underlying the development of cerebellar ataxia in HE remain obscure. Recently, we examined the actions of the CSF obtained from the 6 patients with ataxic HE using patch-clamp recording from the rat cerebellar slices [78]. The CSF from one patient, but not those of the other five patients, impaired the presynaptic short-term plasticity between parallel fiber-Purkinje cell transmissions. This result suggests that defective glutamate release is a potential pathological mechanism in some patients with HE.

In conclusion, cerebellar ataxia is a common presentation in HE, characterized by truncal ataxia, little or absence of cerebellar atrophy on brain imaging and good responsiveness to steroid, which is recognized as “a treatable ataxia”. It is warrant that HE is one of the important differential diagnoses for cerebellar ataxia after carefully excluding other causes.

## Primary Autoimmune Cerebellar Ataxia (M. Hadjivassiliou and P. Shanmugarajah)

In addition to specific cerebellar disease entities where autoimmunity is triggered by another disease (e.g., cancer in paraneoplastic cerebellar degeneration or gluten ingestion in gluten ataxia) there is evidence to suggest that the cerebellum can be a primary organ specific autoimmune disease, hence the proposed term of Primary Autoimmune Cerebellar Ataxia (PACA). This term implies no known trigger factor for the development of immune-mediated damage to the cerebellum a situation analogous to primary hypothyroidism (Hashimoto’s disease), type 1 diabetes mellitus, vitiligo etc.

**Fig. 1:**
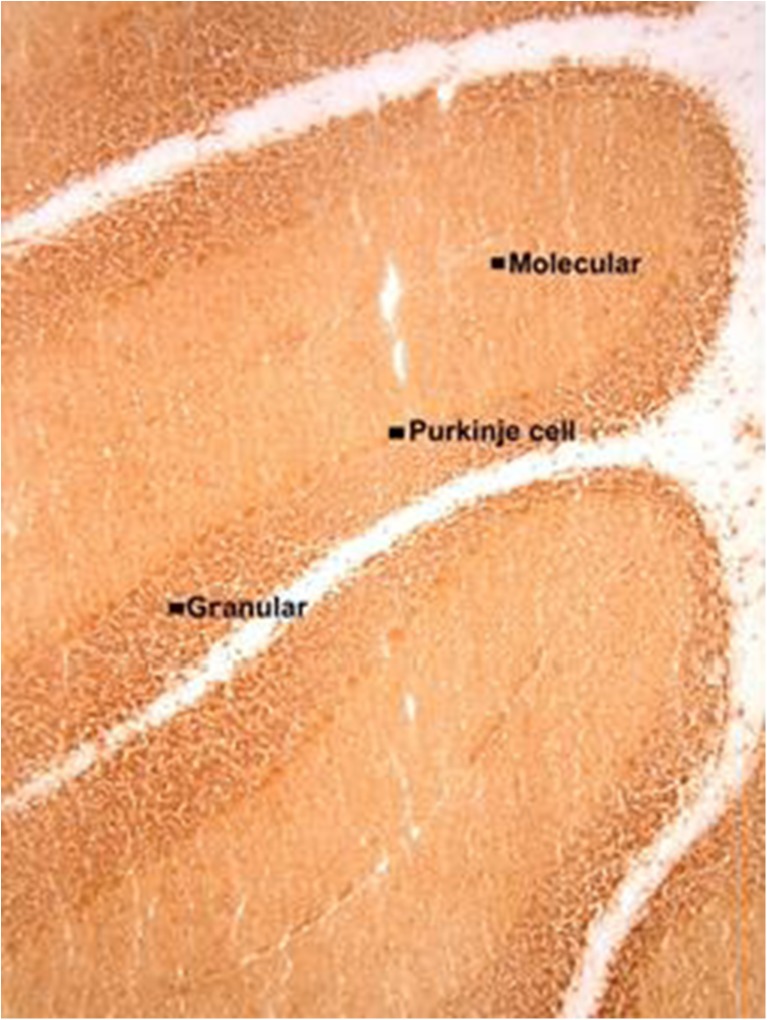
An example of the staining seen using sera (dilution of 1 in 600) from a patient with primary autoimmune cerebellar ataxia on rat cerebellum. There is clear staining of Purkinje cells as well as cells within the granular layer. Such staining is not seen when using sera from healthy controls or patients with genetic ataxia. The serum was negative for all known Purkinje cell antibodies

Evidence in support of such contention is diverse. Firstly, the Human Lymphocyte Antigen (HLA) type DQ2 is significantly overrepresented in patients with idiopathic sporadic ataxia (74 % vs 35 % in the healthy population) whereas the prevalence of this HLA type in patients with genetically characterized ataxias is no different to the one found in the healthy population [9]. The HLA DQ2 has been shown to have a strong association with autoimmune diseases, such as celiac disease and gluten ataxia, type 1 diabetes mellitus, stiff person syndrome, autoimmune thyroid disease and autoimmune polyendocrine syndromes [79–83]. Secondly there is a significantly higher prevalence of one or more autoimmune diseases in patients with idiopathic sporadic ataxia when compared to the general population and to patients with genetic ataxias (47, 3, and 5 %, respectively) [9]. In some cases, such association prompted researchers to attribute the cerebellar dysfunction to the presence of the other autoimmune disease [75]. Thirdly it has been shown that cerebellar antibodies can be present in at least 60 % of patients with idiopathic sporadic ataxia by contrast to 5 % in patients with genetic ataxias [9]. Figure 1 is an example of the staining obtained using serum from a patient with PACA on rat cerebellum. The autoimmune mechanism by which the cerebellum is damaged in the context of autoimmunity remains unclear. The presence of antibodies does not necessarily imply antibody-mediated damage but may prove to be a useful diagnostic aid. As the HLA DQ2 allele is found in up to 35 % of healthy individuals, this test alone cannot serve as a marker for PACA. The presence of additional autoimmune diseases in either the patient or their first-degree relatives may be another helpful pointer. Ultimately, characterization of the cerebellar antibodies may prove to be the best diagnostic marker for PACA.

The next step in unraveling and consolidating PACA as a disease entity is a trial of immunosuppression as a means of treatment. Assessment of the value of immunosuppression in these patients will be challenging. Firstly the choice, timing, and duration of immunosuppression remain unclear. Secondly, monitoring treatment response may prove very difficult because of the variable and mostly slowly progressive nature of the disorder and the crude nature and poor sensitivity of ataxia rating scales. MR spectroscopy of the cerebellum (vermis and hemispheres) is proving to be an important and accurate tool in monitoring disease [84] and this may prove to be a promising way of assessing treatment benefits.

While the final common pathological outcome in PACA, as in other cerebellar ataxias, is the irreversible loss of Purkinje cells, evidence derived from treatment of other immune-mediated ataxias suggests that early intervention may not only stabilize the ataxia but could also salvage malfunctioning cells as evident by the demonstration of clinical and radiological improvement of the ataxia [85].

The clinical characteristics of such patients may also assist in case identification and in some cases distinguish from other causes of ataxia. Patients with PACA tend to develop ataxia in their early 50s. The ataxia in general, tends to be slowly progressive but in a few cases there may be a rather acute onset (a picture not dissimilar to that often seen at presentation in paraneoplastic cerebellar degeneration). In fact some of these patients may have originally been diagnosed as having post infectious cerebellitis, the difference being that they continue to progress rather than improve. This pattern of organ involvement is very similar to what is observed in Hashimoto’s thyroiditis. Subsequently, however such patients follow a much more benign course with a very slowly progressive illness. Additional autoimmune diseases may be already present or may manifest subsequent to the development of the ataxia. Patients with PACA almost always have cerebellar atrophy on MRI but the severity of atrophy depends on disease duration. These patients are also easily distinguished from other patients with sporadic late-onset ataxia such as patients with cerebellar variant of multi-system atrophy by the absence of autonomic involvement and the slower progression.

The next logical step in consolidating this disease entity is an adequately powered study comparing immunosuppressive treatment with placebo. The results of such a study will not only clarify and consolidate the concept of PACA but will hopefully offer hope for a substantial number of patients with progressive sporadic “idiopathic” ataxia.

## Gluten Ataxia (M. Hadjivassiliou)

Gluten ataxia (GA) was originally defined as otherwise idiopathic sporadic ataxia in the presence of circulating anti-gliadin antibodies of IgG or IgA type [8]. This original definition was based on the serological tests available at the time. The development of newer serological markers, more specific to neurological manifestations (anti-TG6 antibodies) may assist in improving the diagnosis but are not as yet readily available [86, 87]. In a series of over 1000 patients with progressive cerebellar ataxia evaluated over a period of 20 years in Sheffield, UK, GA had a prevalence of 15 % among all ataxias and 41 % among idiopathic sporadic ataxias.

GA usually presents with pure cerebellar ataxia primarily affecting gait and lower limbs. Rarely, it can present with ataxia in combination with focal myoclonus [88], palatal tremor [89], or opsoclonus [90]. GA is usually of insidious onset with a mean age at onset of 53 years. Pediatric cases, however, have also been described [91]. Rarely, the ataxia can be rapidly progressive mimicking paraneoplastic cerebellar degeneration. Gaze-evoked nystagmus and other ocular signs of cerebellar dysfunction are seen in up to 80 % of cases. All patients have gait ataxia and the majority have lower limb ataxia. Less than 10 % of patients with GA will have any gastrointestinal symptoms and only 40 % will have evidence of enteropathy on biopsy. As with celiac disease, patients with GA are often found to have an increased prevalence of additional autoimmune diseases the commonest of which include hypothyroidism, type 1 diabetes mellitus and pernicious anemia. Gastrointestinal symptoms are seldom prominent and are not a reliable indicator for the presence or absence of enteropathy. In this respect, gluten ataxia resembles dermatitis herpetiformis, an autoimmune dermatopathy triggered by gluten where gastrointestinal symptoms are not prominent even in the presence of an enteropathy [92].

**Fig. 2:**
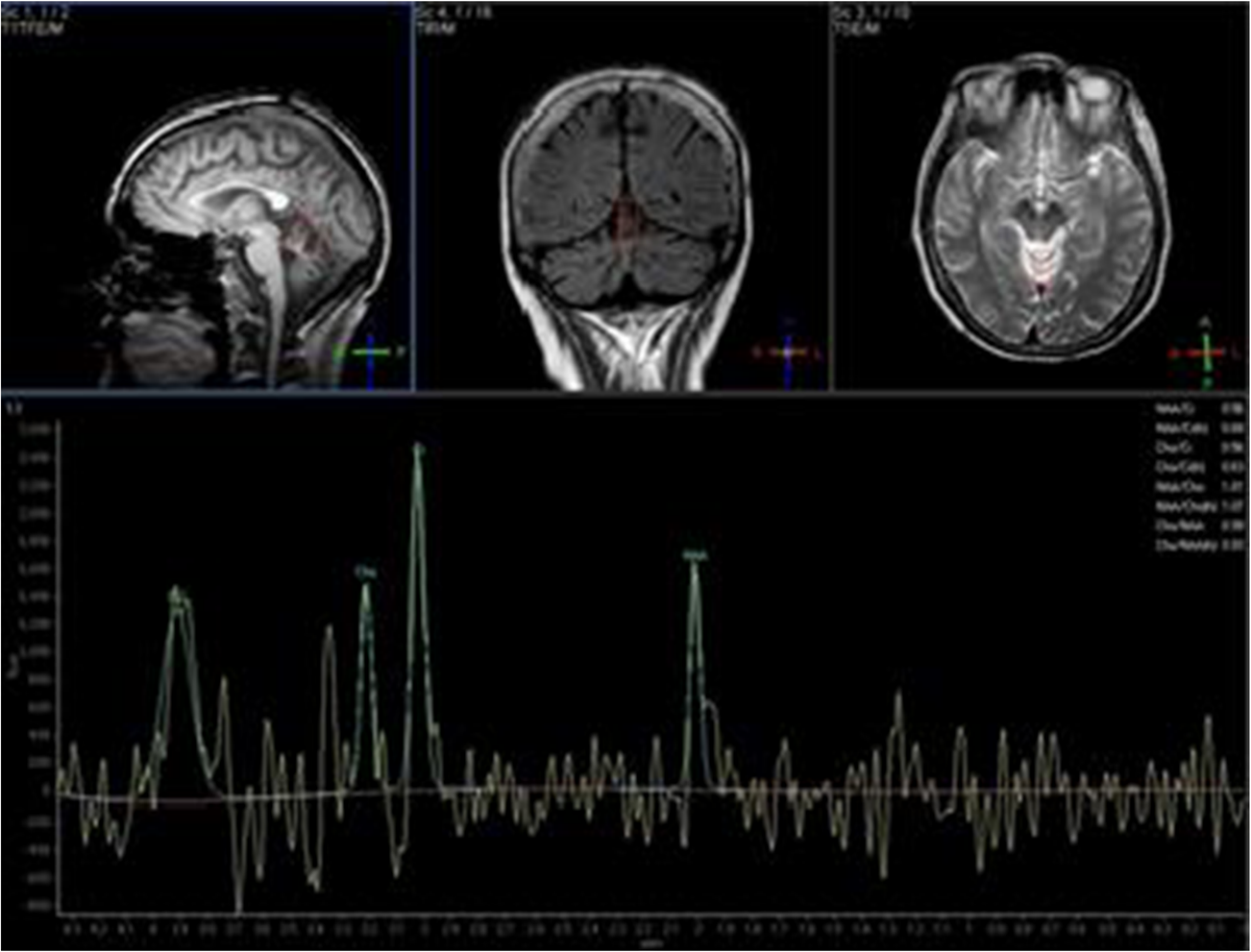
MR spectroscopy of the vermis from a patient with gluten ataxia. There is significant reduction of NAA/Cr ratio (0.56, normally should be above 1). This is a typical finding in patients with gluten ataxia even in the absence of significant atrophy. Primarily involvement of the vermis is also seen in a number of other immune-mediated ataxias in contrast to degenerative and genetic ataxias where there is global involvement of the cerebellum

Up to 60 % of patients with gluten ataxia have evidence of cerebellar atrophy on MR imaging but all patients have spectroscopic abnormalities (reduced *N*-acetylaspartate/creatinine) primarily affecting the vermis (Fig. 2). Recent evidence suggests that patients with newly diagnosed celiac disease (CD) presenting to the gastroenterologists have abnormal MR spectroscopy at presentation and that this is also associated with clinical evidence of cerebellar dysfunction (e.g., nystagmus, gait ataxia) [93]. In addition, voxel-based morphometry and volumetric analysis of the cerebellum reveal significant abnormalities in patients with CD when compared to age-matched healthy individuals [93].

Response to treatment with a gluten-free diet depends on the duration of the ataxia prior to the diagnosis. Loss of Purkinje cells in the cerebellum, the end result of prolonged gluten exposure in patients with GA, is irreversible and early diagnosis and treatment is more likely to result in improvement or stabilization of the ataxia. While the benefits of a gluten-free diet in the treatment of patients with CD and dermatitis herpetiformis have long been established, there are very few studies of the effect of gluten-free diet on the neurological manifestations. Most are case reports primarily concerning patients with established CD who then develop neurological symptoms. Such studies suggest variable but overall favorable responsiveness to a gluten-free diet. A small, uncontrolled study looked at the use of intravenous immunoglobulins in the treatment of four patients with GA without enteropathy [94]. All patients improved. Only one systematic study of the effect of gluten-free diet on a cohort of patients presenting with ataxia, with or without an enteropathy, has been published [85]. This study also reported serological evidence of elimination of the anti-gliadin antibodies as a confirmation of strict adherence to the diet. Forty-three patients with gluten ataxia were enrolled. Twenty six adhered strictly to the gluten-free diet, had serological evidence of elimination of antibodies and comprised the treatment group. Fourteen patients refused the diet and comprised the control group. Patient and control groups were matched at baseline for all variables (age, duration of ataxia, etc.). There was no significant difference in the baseline performance for each ataxia test between the two groups. There was significant improvement in performance in test scores and in the subjective global clinical impression scale in the treatment group when compared to the control group. The improvement was apparent even after excluding patients with an enteropathy. The study concluded that gluten-free diet can be an effective treatment for GA.

Patients with myoclonic ataxia and CD often have refractory enteropathy (non-responsive to the diet) and are at risk of developing enteropathy associated lymphoma [88]. While both the ataxia and enteropathy can be stabilized using immunosuppressive treatment, the myoclonus remains the most disabling feature of this condition which is poorly responsive to symptomatic treatment (e.g., clonazepam, levetiracetam, epilim, piracetam, etc.).

Patients presenting with ataxia due to celiac disease are significantly older from those being diagnosed with CD by the gastroenterologist (mean age 61 vs 47, respectively) [95]. They are often more severely affected by their ataxia. This is also reflected on imaging where the severity of atrophy is marked and the spectroscopy values for *N*-acetylaspartate/creatinine are low.

It is possible that the presence of gastrointestinal symptoms in these patients offers a diagnostic advantage over patients presenting with ataxia by making them more likely to be tested for the disease and hence more likely to be diagnosed and treated early.

## Pathophysiology of Gluten Ataxia (M. Hadjivassiliou and D. Aeschlimann)

There is strong evidence to suggest that the cerebellar insult in GA is immune-mediated. Vitamin deficiencies (e.g., B12, E, copper) are rarely implicated. Postmortem examination from patients with gluten ataxia show patchy loss of Purkinje cells throughout the cerebellar cortex [8, 96, 97]. The cerebellar white matter showed astrocytic gliosis, vacuolation of the neuropil and a diffuse infiltrate mainly of T-lymphocytes. In addition, there was perivascular cuffing with inflammatory cells, mainly T-lymphocytes, with smaller numbers of B-lymphocytes and macrophages.

Experimental evidence suggests that there is antibody cross-reactivity between antigenic epitopes on Purkinje and other cerebellar cells (granular layer) and gluten peptides. For example, anti-gliadin antibodies have been shown to react with human and rat cerebellar Purkinje cells in vitro [98]. However, sera from patients with gluten ataxia appear to contain additional antibodies targeting Purkinje cell epitopes. This is shown by the failure to eliminate such reactivity with Purkinje cells when using sera from patients with gluten ataxia after removal of anti-gliadin antibodies using crude gliadin.

There is increasing evidence of the involvement of transglutaminases in the pathogenesis of GA [99, 100]. Transglutaminase 2, the autoantigen in celiac disease, is one of a family of enzymes that covalently crosslink or modify proteins. Gliadin proteins, the immunological trigger of gluten sensitivity, are glutamine rich donor substrates amenable to deamidation [101]. TG2 may contribute to disease development by deamidating gluten peptides and thereby increasing their reactivity with HLA-DQ2/DQ8 (found in almost 100 % of patients with CD) which potentiates the T cell response. Why should these patients develop autoantibodies to TG2 remains unclear. The formation of complexes of gluten and TG2 may permit gluten reactive T cells to provide the necessary help to TG2-specific B cells. This would also explain why serum antibodies to TG disappear when patients adopt a gluten-free diet. However, it has also been suggested that molecular mimicry, i.e., shared epitopes between deamidated gliadin and TG2, or exposure of masked epitopes upon reaction of the enzyme with antigenic gliadin peptides could lead to autoantibody production [102].

Like TG2 in CD, TG3, an epidermal transglutaminase has been shown to be the autoantigen in dermatitis herpetiformis [103]. Dermatitis herpetiformis is a gluten-sensitive dermatopathy that presents with an itchy vesicular rash and is one example of extra-intestinal manifestations of gluten sensitivity. While the majority of patients with dermatitis herpetiformis have evidence of enteropathy on duodenal biopsy, these patients rarely complain or present with gastrointestinal symptoms. This form of gluten sensitivity may be determined by autoimmunity directed primarily toward TG3 rather than TG2 suggesting that the explanation for the heterogeneity in disease manifestation may lie in the specificity of the immune response. Anti-TG3 antibodies have been shown to induce pathology in an animal model mirroring that of dermatitis herpetiformis, thus further emphasizing the central role that antibodies or antibody complexes play in disease establishment.

In an analogous way, TG6, a brain-expressed transglutaminase has been shown to be a target antigen in patients with GA [86]. Thus, patients with GA have evidence of circulating anti-TG6 antibodies. Such antibodies are gluten dependent and are eliminated by gluten-free diet [87]. TG6 antibodies isolated using phage display methods have been shown to react with both Purkinje cells and cells within the granular layer. Work on brain postmortem tissue of GA patients demonstrate the accumulation of IgA deposits in the cerebellum and brainstem, most prominently within the muscular layer surrounding vessels but also in brain tissue proper [104]. Such intense perivascular changes may imply a perivascular inflammation that compromises blood brain barrier function and allows the entry of gluten related antibodies into the central nervous system. Evidence that such antibodies may potentially be pathogenic comes from mice experiments [98]. Intraventricular injection of phage display produced antibodies against TG2 and TG6 were capable of inducing ataxia in mice within 3 h of injection [99]. A similar observation was made using serum from patients with gluten ataxia [99].

While a lot of insight as to the pathophysiology of gluten ataxia can be gained from the above studies, there are still a number of unanswered questions, namely the reason behind the target of the autoimmune reaction (TG2 vs TG3 vs TG6 or any combination) and ultimately the trigger for the development of symptomatic disease in genetically susceptible individuals.

## Acute Cerebellitis (M. Hadjivassiliou)

Cerebellitis refers to an immune-mediated acute inflammation of the cerebellum. It is generally thought to be the result of an immune response to a recent infection (post infectious cerebellitis) but it may at times represent the onset of primary autoimmune cerebellar ataxia.

There have been numerous reports of acute ataxia due to cerebellitis, predominantly in children, associated with specific infections, commonly viral illnesses such as influenza, parainfluenza, varicella, mumps, measles, rubella, etc. Acute cerebellitis has also been reported with bacterial infections such as pertussus, typhoid, diphtheria, leptospirosis, mycoplasma, Legionaires, etc. Post infectious cerebellitis makes up 50 % of all neurological sequelae of varicella infection and is thus very common in children [105]. In adults, the commonest preceding infection is EBV or mycoplasma [106].

The clinical features are dominated by an acute onset of cerebellar dysfunction, not infrequently associated with some confusion or disorientation. Pediatric cases have a peak incidence at 3 years of age with 34 % having severe ataxia causing inability to walk. The mean latency from onset of prodromal illness to onset of ataxia is 9.9 days. The mean recovery period is about 2 months with the majority of the patients (88 %) making a full recovery [105]. In adult series, the clinical features were similar with the addition of oculomotor disturbances in 73 %. The latency from onset of prodromal illness to development of ataxia was longer at 3.5 weeks. CSF examination shows elevation of white cell count, predominantly lymphocytes, in 50 % of patients and high protein in about 30 % of patients [106]. MRI may demonstrate cerebellar swelling.

In those patients where recovery is incomplete (18 %), there was cerebellar atrophy evident on MRI. These cases may represent a subgroup of patients where acute cerebellitis is a primary autoimmune phenomenon leading to permanent and progressive ataxia, analogous to what is seen in Hashimoto’s thyroiditis that can precede the onset of hypothyroidism [9].

While the use of DNA amplification techniques sometimes reveal evidence of viral products in the CSF it is thought that acute cerebellitis is in fact immunologically mediated. This is supported by the specificity in the involvement of the cerebellum with sparing of other parts of the brain, the presence of oligoclonal bands in some cases and generally good prognosis. One study demonstrated the presence of antineuronal antibodies in acute cerebellitis following EBV infection [107].

Isolated case reports of fatal cerebellitis usually due to severe swelling and brain herniation are rare and the only source of pathology. The neuropathological findings seen in some of these reports are compatible with an acute meningoencephalitis while other reports are more in favor of a post infectious immune reaction similar to acute disseminated encephalomyelitis [108].

## Cerebellar-like Ataxia in Miller Fisher Syndrome and Related Conditions (N. Yuki)

In 1956, Miller Fisher described 3 cases of “An unusual variant of acute idiopathic polyneuritis (syndrome of ophthalmoplegia, ataxia and areflexia)” [109]. In 1962, Richter described the “ataxic form of polyradiculoneuritis (Landry-Guillain-Barré syndrome)” characterized in a patient with profound ataxia without weakness, no or minimal ophthalmoplegia, no sensory loss (particularly for proprioception), and without a Romberg sign [110]. The clinical presentation of ataxic Guillain-Barré syndrome is very similar to Miller Fisher syndrome except for the absence of ophthalmoplegia. In 1992, the seminal work by Chiba and colleagues in discovering anti-GQ1b IgG antibodies in patients with Miller Fisher syndrome led to the unraveling of the pathogenesis of Miller Fisher syndrome and related conditions [111]. Patients with ataxic Guillain-Barré syndrome, as well as those with Miller Fisher syndrome, have been found to carry anti-GQ1b IgG antibodies, suggesting that ataxic Guillain-Barré syndrome is an incomplete form of Miller Fisher syndrome [112].

Anti-GQ1b antibodies are likely to have a pathogenic role in the development of Miller Fisher syndrome and ataxic Guillain-Barré syndrome based on the following findings: (i) Antecedent infectious agents such as *Campylobacter jejuni* and *Haemophilus influenzae* bear a GQ1b epitope [113, 114]; (ii) GQ1b is strongly expressed in the oculomotor nerves, large-diameter dorsal ganglion neurons and muscle spindles [115–117]; (iii) Human anti-GQ1b antibodies in the presence of complement kill dorsal root ganglion neurons in rats [118].

The site and nature of the pathophysiology of ataxia in Miller Fisher syndrome have yet to be established because there have been no autopsy reports of pure Miller Fisher syndrome. However, repeated nerve conduction studies have shown that, at the least, the peripheral sensory nerve is one of the sites, and the nature of the damage is axonal, not demyelinating [119]. Sensory nerve action potential amplitudes can recover rapidly. This pattern of rapid reversibility, occurring within weeks and without demyelinating features, suggests dysfunction of the nodal and paranodal regions. In other patients, slow progressive improvement or persistent changes in sensory nerve action potential amplitudes represent axonal degeneration. Based on the patterns of sensory abnormality, the antigen target of the autoantibody attack may be in the sensory nerves. Reversible conduction failure, as demonstrated in cutaneous afferents, is unlikely to be responsible for the prominent ataxia in Miller Fisher syndrome. However, reversible conduction failure may occur also in Ia afferents, which are difficult to investigate in routine nerve conduction studies. This may explain the rapid and generally complete recovery of ataxia in Miller Fisher syndrome, which would be incompatible with the exclusive involvement of dorsal root ganglia.

Some patients with Miller Fisher and Guillain-Barré syndromes show striking limb incoordination with jerky rhythmical features not influenced by eye closure, no objective sensory changes, and a negative Romberg test. Although neither nystagmus nor cerebellar dysarthria were present, this form of ataxia seemed to be of cerebellar origin [109, 110]. However, Miller Fisher himself postulated that the “cerebellar-like” ataxia in these patients was of peripheral origin due to a “unique widespread and selective attack on the sensory neurons underlying postural adjustment” [109]. The only autopsy study of ataxic Guillain-Barré syndrome showed no lesions in the cerebellum but major degeneration of the fibers of the posterior and Clarke columns without involvement of the neurons of Clarke columns [110]. This suggested that “cerebellar-like” ataxia is a consequence of the damage of afferent fibers to the spinocerebellar system, although neither nerves nor dorsal root ganglia had been sampled in the autopsy study. Postural body sway analysis indicated selective involvement of the Ia afferent system group in a patient with ataxic Guillain-Barré syndrome who had anti-GQ1b antibodies, as well as in patients with Miller Fisher syndrome [120, 121]. These findings suggest a dysfunctional proprioceptive afferent system and that cerebellar-like sensory ataxia is caused by selective involvement of muscle spindle afferents, as also demonstrated by the absence of the H-reflex with normal sensory conduction and normal sensory-evoked potentials.

Anti-GQ1b antibodies stain neural components and intrafusal muscle fibers of spindles in mice, rats, and humans [117], and mostly stain parvalbumin-positive nerves and putative nerve endings in rat muscle spindles, which provide evidence that proprioceptive nerves highly express GQ1b [122]. IgG with anti-GQ1b reactivity from a patient with Guillain-Barré syndrome bound to the nodes of Ranvier in human dorsal roots [123]. Antibodies to GQ1b also stain some large neurons in human dorsal root ganglia [116]. MRI has shown enhancing lesions in the spinocerebellar tracts at the level of the lower medulla in a patient with Miller Fisher syndrome overlapped by Guillain-Barré syndrome [124]. Overall, these findings suggest that *cerebellar-like* ataxia described in some Miller Fisher syndrome and ataxic Guillain-Barré syndrome patients may be caused by selective involvement of group Ia afferents along their path from muscle spindles to spinal cord, or involvement of the proprioceptive spinocerebellar pathway.

## Paraneoplastic Cerebellar Degenerations (B. Joubert and J. Honnorat)

Paraneoplastic neurological syndromes (PNSs) are rare neurological diseases representing a remote effect of a cancer [125]. By definition, they cannot be explained by metastatic, metabolic, iatrogenic, or infectious causes. Paraneoplastic cerebellar degenerations (PCDs) are the most frequent PNS, making up to 24 % of the reported PNSs [126].

Patients with PCDs present with symmetrically distributed static and kinetic cerebellar symptoms. Symptoms usually install subacutely in a few weeks or months, although fast, stroke-like presentations may as well occur. Extracerebellar symptoms can be occasionally present, expressing additional involvement of limbic regions, brainstem, spinal cord or dorsal root ganglia [127], but cerebellar ataxia is isolated in most of the cases. In 84 % of the patients, the neurological syndrome precedes the discovery of the underlying tumor [5, 127]. CSF analysis may display mild lymphocytosis, increased proteins, and/or oligoclonal bands. Brain MRI is usually normal at the onset of the disease and cerebellar atrophy, preferentially involving the vermis, may appear only at later stages. Alternative diagnoses must be cautiously excluded. A cerebellar tumor, stroke, or a metabolic disease are usually easily ruled out, while leptomeningeal metastasis, late-onset degenerative cerebellar atrophy, infectious cerebellitis, and chemotherapy adverse effects can be difficult to distinguish from a paraneoplastic etiology.

Most PCDs associate with the so-called onconeural antibodies (ONA), such as anti-Hu, anti-Yo, anti-CV2/CRMP5, anti-Ma2, anti-Ri, or anti-Tr/DNER antibodies [127] (Table 3). Those autoantibodies target intracellular neuronal antigens that are ectopically expressed by the tumor [128]. They are found in 80 % of PCDs patients, emphasizing the probable autoimmune origin of this disease [5]. Most rarely PCDs have been described in patients with SCLC and anti-VGCC antibodies.

A cancer is found in up to 84 % of ataxic patients with serum ONA, gynecologic cancers (breast, ovaries, uterus), small cell lung carcinoma and Hodgkin’s lymphoma being the most frequent [127]. Each autoantibody associates with specific tumor types [127] and therefore the nature of the antibody guides the search for the causative cancer (Table 2). Whole body Computed tomography (CT) scan, pelvic ultrasonography, and mammography are essential first-line tests. Whole-body FDG positron photon emission computed tomography (PET) scanner examinations greatly improve cancer detection in PNSs [129]. In rapidly evolving and severe cerebellar ataxia, exploratory laparotomy in women with anti-Yo antibody, or orchidectomy in male patients with anti-Ma2 antibodies, may be necessary in order to detect microscopic tumors [130, 131]. If inconclusive, investigations must be repeated at a regular basis.

The pathophysiological function of ONA is still under debate. They may not play a role by themselves but probably only act as a biological marker of the autoimmune process. The observed Purkinje cells death is supposed to be mainly due to a cell-mediated cytotoxic immune response. Indeed, circulating activated CD8+ T cells specific for Yo or HuD antigens have been found in PCDs patients with such autoantibodies [132]. Brain biopsies and autopsies have shown the presence of CD8+ T cells infiltrates and activated microglia in the cerebellum associated to a profound loss of Purkinje cells, while B cells, IgG, or complement could not be detected [133]. On the other hand, anti-Yo antibodies incubated ex vivo with cerebellar slices are actively internalized by Purkinje cells, and significantly increase neuronal death, suggesting that those specific autoantibodies may play some role in the disease [134]. Nevertheless, various experiments, including the injection of patients’ antibodies, the immunization of murine model with purified Yo or HuD antigens, and activated T cells injections failed to create an animal model of PCDs [135, 136].

Patients’ outcome is usually poor. Due to the diffuse neuronal loss, neurological improvement is rare, and treatments allow only for a stabilization of the symptoms. Tumor removal and physiotherapy are currently the cornerstone of the treatment. Immunotherapy is often disappointing, likely due to the early neuronal death. Because of the rarity of PCDs, there is to date no large-scale clinical study in this field. A few retrospective studies and case reports have suggested a beneficial effect of corticosteroids and intravenous immunoglobulins (IvIg) in some patients with anti-Hu or anti-Yo antibodies. Therefore, there may be subgroups of patients with a better response to immunotherapy, although their distinctive features remain to be defined. Prognosis also varies depending on the nature of the associated ONA. For instance, anti-Tr associated PCD are particularly remarkable for the improvement observed after efficient treatment of the associated lymphoma, irrespective of additional immunotherapy [137].

It has to be noted that cerebellar ataxia is exceptionally associated with antibodies targeting synaptic antigens, such as the metabotropic glutamate receptor mGluR1 [138]. In such cases, tumors are infrequent and immunotherapy is usually efficient, due to the fact that symptoms are likely due to a direct and reversible functional effect of the autoantibodies themselves on synaptic transmission.

Future studies are needed in order to clarify the respective role of autoantibodies and cytotoxic T cells in the development of PCDs, as well as to define optimal therapeutic strategies for this debilitating disease.

## Ataxia Associated with SLE (P. Chattopadhyay and K. Adhikari)

In lupus, the most common cause of acute ataxia is damage to cerebellum either due to the disease activity itself as part of neuropsychiatric systemic lupus erythematosus (NPSLE) or can be secondary due to acute disseminated encephalomyelitis, drug-related inflammatory damage, vasculitis or atypical infections [139, 140]. Secondary causes are twice as common as primary NPSLE and the treatment strategies are very different [141].

Cerebellar involvement in SLE is rare occurring in less than 2 % of cases [142–148]. Like in multiple sclerosis, the immunological mechanisms responsible are not specific to the cerebellum and usually affect other parts of the central and peripheral nervous system. Females in reproductive age group are primarily involved. The clinical features of ataxia depend on the pathogenic mechanism as well as the part of cerebellar and its connection involved. Acute ataxia in lupus may be either a focal involvement- due to vaso-occlusion due vasculopathy (mainly small vessel), vasculitis, leuco-aggregation or thrombosis resulting in ischemia, infarction, hemorrhage or vasogenic edema; or a diffuse cerebellar involvement- due to different antibodies such as anti-DsDNA, cross reacting to brain NMDA receptors mediating cerebellar injury or dysfunction. Other causative factors may be excitotoxicity, diffuse endothelial injury, and leucothrombosis in microvasculature mimicking Schwartzmann phenomenon, resulting from intravascular complement activation leading to leucocytes and platelet adhesion with endothelium [149]. Focal involvement present with ipsilateral limb and ocular ataxia with cerebellar hemispheric signs, often with neurologic signatures of associated brainstem involvement. Diffuse involvement on the other hand present with acute/subacute pancerebellar symptoms of bilateral limb, gait, and ocular ataxia often in isolation without other supporting neurologic findings [147, 148].

Thromboembolism and hypercoagulability resulting in microinfarcts and small vessel vasculopathy is the most common pathological substrate of NPSLE. Macroinfarcts, micro or macrohemorrhages, cortical atrophy, demyelination, true vasculitis has also been described [149, 150].

MRI is the investigation of choice [140]. Conventional MRI may reveal diffuse cerebellar atrophy and volume loss along with diffuse cerebral, corpus callosal and hippocampal atrophy, although periventricular and subcortical white matter lesions indicative of edema and small infarct are most common MRI findings. Data related to cerebellar involvement in lupus are sparse though cerebellar changes are associated with neurocognitive deficits in other inflammatory and ischemic diseases. In pediatric lupus patients, cerebral and cerebellar volume loss is maximum in patients with NPSLE in the first 4 years of disease presentation [151]. Cerebellar, cerebral, and corpus callosal atrophy are present in newly diagnosed patients with NPSLE, possibly indicating sequel of active neurolupus rather than effect on cumulative steroid dosage. Patients with previous nephritis have less prominent volume loss indicating control of neuro-inflammation as these patients have already received immunosuppressive therapy [151]. Functional studies such as PET or single photon emission computed tomography (SPECT) scan may reveal patchy areas of dysfunction in brain areas unaffected in conventional MRI suggesting an uncoupling of cerebellar metabolism unrelated to obstruction of blood flow.

Management will be expectant with minimum intervention in mild CNS disease, as opposed to focal or diffuse severe CNS syndromes which require urgent intervention. Symptomatic therapy, anticoagulants, and immunosuppressive treatments are cornerstones of therapy. Apart from different stroke episodes, which require either stroke prevention with antithrombotic therapy or control of hypertension, other acute cases of cerebellar ataxia often require immunologic modification mainly augmentation of steroid and or intravenous cyclophosphamide therapy. High-dose intravenous methyl prednisolone therapy is often effective, reversing the MRI abnormality followed by clinical improvement [148].

## Reversibility of Immune-Mediated Cerebellar Ataxia: How Impaired are the Cerebellar Predictive Control Mechanisms? (S. Kakei, J. Lee, K. Nanri and H. Mizusawa)

When we make a reaching movement, there are infinite possible combinations of muscle activities that could produce the same movement kinematics, due to the redundancy of the musculo-skeletal system (see, e.g., [152] for a review). Nevertheless, muscle activities are usually stereotyped, suggesting that there is a hidden constraint why the controller chooses a particular pattern. The constraint, if identified in both health and neurological disorders, could provide valuable information to provide pathophysiological explanations for movement disorders. Therefore, it is desirable to develop a method to functionally characterize diverse patterns of muscle activities [153, 154].

Recently, we developed a novel method to characterize patterns of muscle activities in terms of their similarities to components of movement kinematics [154]. In this method, we determine a symmetric relationship between the linear sum of activities of the four wrist prime movers and the second-order linear equation of motion for the wrist joint in terms of the joint torque. The symmetric relationship determines a set of parameters that characterize the muscle activities by quantifying their similarity to the components of movement kinematics of the wrist joint. Recently, we applied this method to compare functional characteristics of muscle activities of immune-mediated CAs, degenerative CAs, and age-matched normal controls.

The six patients with immune-mediated CAs (three patients with anti-GAD-Ab associated CA, two with gluten ataxia, and one patient with cerebellar type of Hashimoto’s encephalopathy) included three males and three females, with a mean age of 66.6 ± 7.2 years (±SD), with a mean disease duration of 6.2 ± 6.4 years. With regard to activities of daily living, three patients walked without assistance while three used walkers. These patients showed clinical improvement following immunotherapy. The ICARS of 23.4 ± 15.5 improved to 14.8 ± 13.6. Two patients exhibited distinct cerebellar atrophy. Whereas three patients showed mild cerebellar atrophy and the cerebellum was normal in one patient. Furthermore, these four patients showed no obvious atrophy of the white matter on MRI voxel-based morphometry. The study also included eight patients with degenerative CAs (five patients with multiple system atrophy and three patients with spinocerebellar ataxia 6; four males and four females, mean age, 64.2 ± 10.5 years), with a mean disease duration of 4.8 ± 2.3 years. Five of these patients walked without assistance, two used walkers, and one patient was wheelchair-dependent. The present study also included nine normal control subjects (six males and six females, mean age, 61.9 ± 8.8 years) free of neurological abnormalities.

We asked the subjects to perform a smooth tracking movement of the wrist joint with a manipulandum (Fig. 1a in [154]). They were required to maintain the position of the cursor within a target moving smoothly along a path of the Fig. 2 at a constant slow speed [154]. We evaluated accuracy of movement with a tracking score. The tracking score represents how much time (in %) the subject keeps the cursor within the target from start to the end of a trial. During the task, we recorded the wrist position (*X* and *Y*) and surface EMG signals of the four wrist prime movers (extensor carpi radialis, extensor carpi ulnaris, flexor carpi ulnaris, and flexor carpi radialis muscles) at 2 kHz [154].

We approximated a relationship between EMG signals of the four wrist prime movers and movement kinematics with the Eq. (1) (simplified from [154]).

1 $$ \tau (t)={\displaystyle \sum_{i=1}^4{a}_i{T}_i(t)=M\ddot{\theta}(t)+B\dot{\theta}(t)+K\theta (t)} $$

where, *τ*(*t*) denotes the wrist joint torque calculated from the wrist joint kinematics. *T*_*i*_ represents the tension of each muscle and *a*_*i*_ denotes parameters that convert the muscle tension into the wrist joint torque. The variables *θ*(*t*), $\dot{\theta}(t)$ and $\ddot{\theta}(t)$ represent angle, angular velocity, and angular acceleration of the wrist joint. *M*, *B*, and *K* represent the inertia [kg m^2^], the viscous coefficient [N m s/rad] and the elastic coefficient [N m/rad] for the wrist joint. We searched for the combination of *B* and *K* that yielded the best match for the Eq. (1). Importantly, the best symmetric relationship was obtained when *B* was in a specific ratio to *K* (see Fig. 4 in [154]). Thus, we used the *B*/*K* ratio as a parameter to characterize the muscle activities in terms of the movement kinematics.

**Fig. 3:**
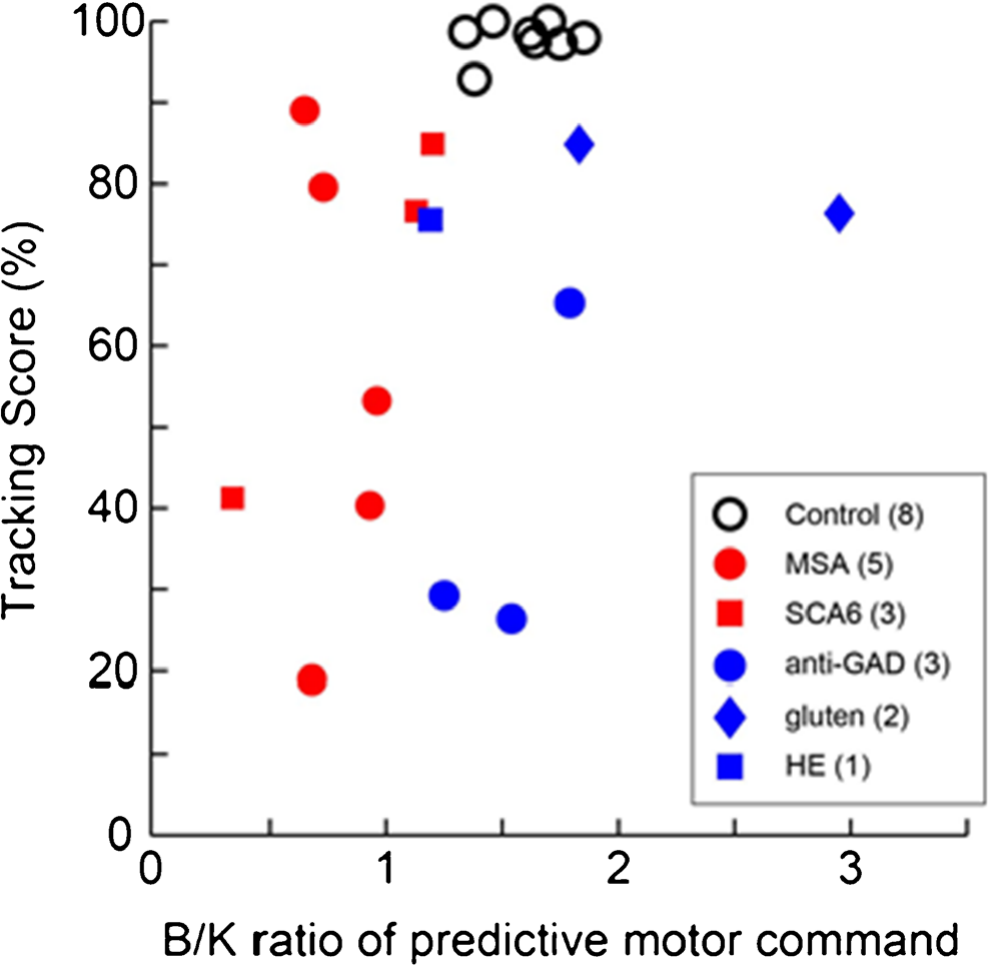
Relationship between the B/K ratio of predictive motor command and tracking score. Circles represent the normal control subjects. *Red symbols* represent the patients with degenerative CAs. *Blue symbols* represent the patients with immune-mediated CAs. *MSA* multiple systemic atrophy, *SCA6* spinocerebellar ataxia type 6, *anti-GAD* anti-glutamic acid decarboxylase (GAD) antibody associated cerebellar ataxia, *gluten* gluten ataxia, *HE* cerebellar type of Hashimoto’s encephalopathy

We calculated the *B*/*K* ratio to characterize the muscle activities in terms of the movement kinematics for the normal controls, the patients with degenerative CAs and the patients with immune-mediated CAs. The control subjects were capable to follow the moving target smoothly with high accuracy (tracking score 93–100 %, *m* ± sd = 97.8 ± 2.3, *n* = 8). Their *B*/*K* ratios ranged from 1.34 to 1.85 (*m* ± sd = 1.59 ± 0.18, *n* = 8) (Fig. 3), suggesting that their muscle activities were weighted comparably or more for velocity control than for position control of the wrist joint. In contrast, *B*/*K* ratios for the degenerative CA patients were much lower (0.68–1.20, 0.83 ± 0.28, *n* = 8) (*p* < 1.4 × 10^−5^, Student’s *t* test), suggesting that the muscle activities of the degenerative CA patients contained much less component for velocity control. The lower weight for smooth velocity control may explain rough movement kinematics (Fig. 4 in [154]) and poor accuracy (Fig. 3) (19–89 %, 60.5 ± 25.6, *n* = 8) (*p* < 0.001) of the tracking in the patients with degenerative CAs. On the other hand, for the patients with immune-mediated CAs, accuracy of tracking was as poor (26.4–84.9 %, 59.68 ± 29.4, *n* = 6) (*p* > 0.95) as the patients with degenerative CAs (Fig. 3). Nevertheless, their *B*/*K* ratios were comparable or even higher (1.19–2.95, 1.75 ± 0.64, *n* = 6) (*p* > 0.49) than that of normal controls, suggesting that ability to generate muscle activities for velocity control was more preserved in the patients with immune-mediated cerebellar ataxia than in the patients with degenerative CAs.

In the task employed in this study, it was necessary to control both velocity and position of the wrist joint to track the moving target accurately. In accordance with this requirement, both velocity and position were *comparably* encoded in the muscle activities of the normal controls as was represented in their *B*/*K* ratios (Fig. 3, open circles). In contrast, for the patients with degenerative CAs, *B*/*K* ratios were significantly lower than the normal controls, suggesting a relative lack of velocity control (Fig. 3, red symbols). Consequently, tracking accuracy of the degenerative CA patients was much poorer than the control subjects (Fig. 3). On the other hand, in the case of the patients with immune-mediated CAs, their movement kinematics appeared as ataxic as that of the patients with degenerative CAs. Nevertheless, their *B*/*K* ratios remained comparable to those of the normal controls (Fig. 3, blue symbols). How can we interpret the striking contrast of *B*/*K* ratios in the two groups of patients with ataxia? In degenerative CA patients, the relative lack of velocity control is basically irreversible due to degeneration of cerebellar neurons. On the other hand, in patients with immune-mediated CAs, the velocity control appears to remain functional at least in early phase, although its accuracy is deteriorated considerably. Furthermore, a part of the deficit appears to remain reversible because they are sometimes treatable [32, 49, 61, 155, 156]. The contrasting changes in B/K ratios for the immune-mediated CAs and the degenerative CAs might be explained as follows. In patients with degenerative CA, outputs from Purkinje cells (PCs) are lost due to severe loss of PCs. By contrast, in the patients with immune-mediated CAs, outputs from PCs are inadequately augmented, at least initially, due to blockade of strong GABAergic inhibition from molecular layer interneurons (most notably basket cells) [61]. In both conditions, the cerebellum might well generate erroneous predictions because of insertion of virtual errors [157], although in different manners. In conclusion, analysis of *B*/*K* ratio in ataxic patients may provide a unique and useful clue to find potentially treatable ataxias.

## Conclusion (H. Mitoma)

In this consensus paper, we have attempted to capture the diversity of the clinical features and proposed pathological mechanisms of immune-mediated cerebellar ataxias (CAs), by gathering contributions from an international panel of experts. The main points of the consensus based on various areas of discussion of immune-mediated ataxias include the following:

### Clinical Features

In addition to the classical immune-mediated diseases (paraneoplastic cerebellar degeneration, Miller Fisher syndrome), the clinical entity of nonparaneoplastic immune-mediated CAs has been established recently. This includes anti-GAD-Abs associated CA, cerebellar type of Hashimoto’s encephalopathy, primary autoimmune cerebellar ataxia (PACA), and gluten ataxia. The clinical features of these diseases are summarized in Table 3.

**Table 3:** Frequency, clinical features and cancer associations of PCDs associated onconeural antibodies

ONA status	Anti-Yo	Anti-Hu	Anti-CV2	Anti-Ri	Anti-Tr	Anti-Ma2	Anti-VGCC	Anti-SOX1	Anti-ZIC4	Seronegative
Frequency among PCDs (%)	53	15	4	2	5	2	2	–	–	18
Associated tumors	Breast Uterus Ovaries	SCLC	SCLC, thymoma	Breast	Hodgkin’s disease	Testicle, lung	SCLC	SCLC	SCLC	Lung, genito-urinary, breast, lymphoma
Extracerebellar symptoms	–	PEM, SN	Neuropathy, uveitis, LE	OM	–	Brainstem (diplopia, dysphagia, dysarthria), LE	LEMS	LEMS	LEMS	–

Frequency among PCDs was evaluated based on the report by Shams’ili et al. [127]

*SCLC* small cell lung cancer, *PEM* paraneoplastic encephalomyelitis, *SN* sensory neuronopathy, *LE* limbic encephalitis, *OM* opsoclonus-myoclonus, *LEMS* Lambert-Eaton myasthenic syndrome

### Diverse Pathomechanisms

Vasculitis is presumed to be the main pathological mechanism of Hashimoto’s encephalopathy and ataxia in systemic lupus erythematosus. In paraneoplastic cerebellar degeneration, cell-mediated autoimmunity is likely responsible for CAs, and autoantibodies are considered to be the marker, not the pathogenic agent. On the other hand, the pathogenic roles of autoantibodies have been suggested in anti-GAD-Abs associated CA and Miller Fisher syndrome, and probably also in gluten ataxia. Taken together, it is possible that various forms of autoimmunity operate in immune-mediated diseases, resulting in the development of CAs. Disease-specific mechanisms of the autoimmunity require further clarification.

### Reversibility and Treatable ataxias

**Fig. 4:**
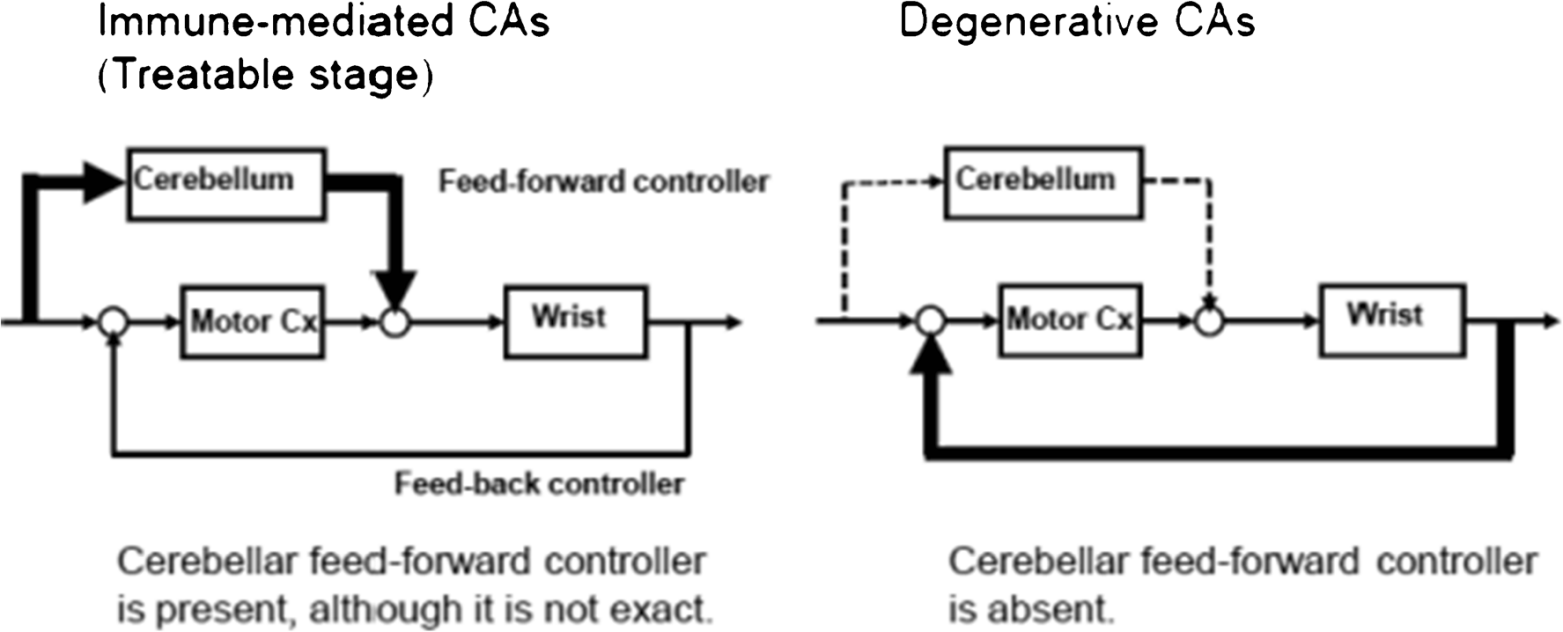
In immune-mediated cerebellar ataxias (*CAs*), cerebellar feed-forward control is still present, although its property is not exact, whereas in spinocerebellar degeneration (SCD), cerebellar feed-forward control is abolished and, instead, feed-back control is compensatively operational. The survival of cerebellar feed-forward controls could be a physiological evidence for reversibility in immune-mediated CAs. *Motor Cx* Motor cortex

By using recent advances in physiological methods, the ratio of feed-forward or feed-back controls can be determined. The operation of feed-forward control could be an index for the survival of cerebellar function. Interestingly, the motor control in patients with immune-mediated CAs was in contrast to that in patients with degenerative CAs, although both patients similarly showed clumsiness in tracking tasks. In the former, feed-forward control is still present, although its property is not exact, whereas in the latter feed-forward control is abolished and, instead, feed-back control is compensatively operational (Fig. 4). The survival of feed-forward controls could be a physiological evidence for reversibility and treatable feature of immune-mediated CAs, suggesting the importance of early diagnosis and immune therapy.

Historically, the establishment of the new disease entity has highlighted the hidden role of the cerebellum in motor control. In the beginning of twentieth century, differentiation of degenerative CAs, e.g., olivo-ponto-cerebellar atrophy, from dorsal column ataxias led to the beginning of neurology of the cerebellum. After World War I, comprehensive studies on trauma of the cerebellum widened the understanding of coordination and dysmetria. Similarly, further studies on immune-mediated CAs might open a new page of cerebellar symptomatology, especially about how the acquired functions by the cerebellum are disrupted.
